# Potential contribution of increased soluble IL-2R to lymphopenia in COVID-19 patients

**DOI:** 10.1038/s41423-020-0484-x

**Published:** 2020-06-25

**Authors:** Yaguang Zhang, Xiaojing Wang, Xuezhen Li, Dong Xi, Ruizhi Mao, Xiaohui Wu, Shipeng Cheng, Xiaoyu Sun, Chunyan Yi, Zhiyang Ling, Liyan Ma, Qin Ning, Yiru Fang, Bing Sun, Di Wu

**Affiliations:** 1 State Key Laboratory of Cell Biology, CAS Center for Excellence in Molecular Cell Science, Shanghai Institute of Biochemistry and Cell Biology, Chinese Academy of Sciences, 320 Yueyang Road, 200031 Shanghai, China; 2 Department and Institute of Infectious Disease, Tongji Hospital, Tongji Medical College, Huazhong University of Science and Technology, 430030 Wuhan, China; 3 Clinical Research Center and Division of Mood Disorders, Shanghai Mental Health Center, Shanghai Jiao Tong University School of Medicine, 200030 Shanghai, China

**Keywords:** Cytotoxic T cells, Infection

Since the outbreak of coronavirus disease 2019 (COVID-19) caused by severe acute respiratory syndrome coronavirus 2 (SARS-CoV-2), more than 6 million cases are confirmed and over 300,000 cases are dead after infection. Dysfunction of immunity in COVID-19 patients has been considered as one of the fatal factors for patients, especially cytokine release syndrome and lymphopenia.^1–3^ The reduced number and increased exhaustion level of lymphocyte are associated with elevated inflammatory cytokines in COVID-19 patients.^4,5^ However, the mechanism of cytokine-induced lymphopenia in COVID-19 is very unclear. IL-2 is critical for the proliferation, differentiation, and function of T cells, including Tregs, CD4^+^, and CD8^+^ effector cells.^6^ Here, we reported the negative relationship between the concentration of soluble IL-2 receptor (sIL-2R) and T-cell number in blood from COVID-19 patients. In vitro addition of recombinant CD25 could inhibit the proliferation and function of T cells from PBMC after stimulated with TCR signaling, which could be rescued by strong IL-2 signaling. Our data suggested the importance of IL-2 signaling in lymphopenia of COVID-19 patients.

In the previous report,^7^ we have reported that plasma cytokines including sIL-2R, IL-6, TNF-a, and IL-10 concentrations on admission were significantly higher in severe cases than moderate cases. To investigate the correlation between plasma cytokines and lymphocytes in blood from COVID-19 patients, the data that has a one -to-one relationship between the cytokines and lymphocytes from 11 samples of 9 patients were analyzed. Among those patients, two patients (patient 8 and patient 9) were from the patients included in our previous report.^7^ The remaining seven patients (from patient 1 to patient 7) with available dynamic data of cytokines and lymphocytes subset counts were consecutive patients admitted from February 18–22, 2020. Samples were collected as shown in Supplementary Fig. 1a.

We divided the samples into two groups (one group contained samples collected within 10 days after illness onset and another group contained samples collected between 10 and 20 days after illness onset). We found that the longer it took after illness onset, the less percentage of CD3^+^ and CD8^+^ T cells was (Fig. 1a). HLA-DR molecules are important surface activation markers for T cells. We also found the impaired HLA-DR^+^ CD3 T cells in long-term onset group (Fig. 1a), whose CD4^+^ T cells, CD19^+^ B cells and NK cells percentage were not reduced when compared with short-term onset group (Supplementary Fig. 1b). Through analyzing the time axis, we found that sIL-2R was positively related to days after illness onset, but not TNF-a, IL-10 and IL-6 (Fig. 1b and Supplementary Fig. 1c). The sIL-2R (including 3 subunits of IL-2R: CD25, CD122, and CD132) was measured using the sandwich enzyme-linked immune-sorbent assay (ELISA). The concentration of sIL-2R in plasma from COVID-19 patients was much higher than healthy controls (Fig. 1c).

**Fig. 1:**
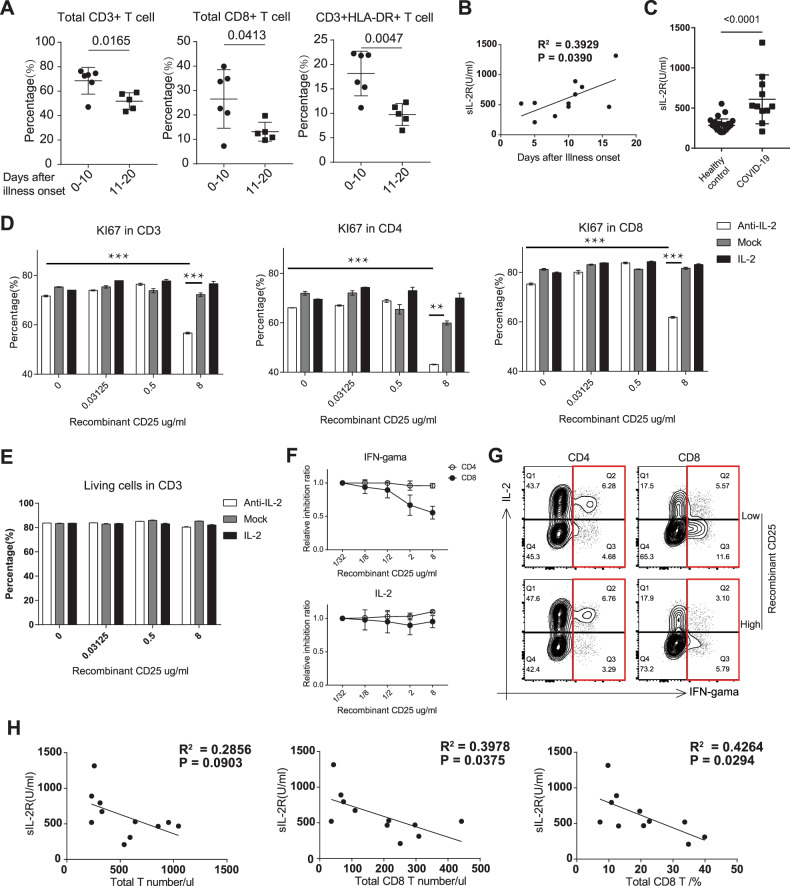
Potential contribution of increased soluble IL-2R to lymphopenia in COVID-19 patients. **a** Percentage of CD3^+^, CD8^+^ and CD3^+^HLA-DR+ T cells in blood from COVID-19 patients within different days after illness onset. The statistical method is the Student’s *t* test. **b** The correlation analysis between soluble IL-2 receptor (sIL-2R) and days after illness onset in COVID-19 patients. **c** The concentration of sIL-2R in plasma from healthy controls (n = 28) and COVID-19 (n = 11). The correlation was assessed with Pearson’s test. Flow cytometric analysis of KI67 (**d**) and cell death with Fixable Viability Dyes (**e**) in T cells after anti-CD3 and anti-CD28 antibodies activation for 3 days within different concentrations of recombinant CD25 and different intensities of IL-2 signaling.***P < 0.001; **P < 0.01 (one-way ANOVA with Tukey’s multiple comparisons test). Recombinant CD25 mediated inhibition rate (**f**) and percentage (**g**) of IFN-gamma in T cells after anti-CD3/28 antibodies activation for 18 h within different concentrations of recombinant CD25 and different intensities of IL-2 signaling. **h** The correlation analysis between soluble IL-2 receptor (sIL-2R) and T cell number in COVID-19 patients. The correlation was assessed with the Pearson’s test

Circulating sIL-2R has been shown to regulate T-lymphocyte activation in various immunological disorders and increased sIL-2R concentration in plasma predicts a decreased cellular response to IL-2.^8^ Serum levels of sIL-2R are significantly higher in Kawasaki disease patients,^9,10^ who suffer a systemic inflammatory disease closely associated with infections.^11^ The latest clinical findings that a pediatric patient diagnosed and treated for Kawasaki disease in the setting of confirmed COVID-19 infection indicate the underlying connection between the two diseases and the potential function of sIL-2R in COVID-19.^12^

Combined with the important function of IL-2 signaling in T cells, we speculated the important function of sIL-2R during the onset of lymphopenia in COVID-19 patients. Induced Ki67 expression stimulated with anti-CD3/28 antibodies among CD3^+^/CD4^+^/CD8^+^ T cells in PBMC from a healthy donor was decreased in the high concentration of recombinant CD25, which could be rescued by strong IL-2 signaling (Fig. 1d, S-Fig. 1d, e). However, recombinant CD25 mediated IL-2 signaling inhibition was not involved in cell death in T cells (Fig. 1e). Flow cytometric analysis showed that IFN-gamma, but not IL-2 expression in T cells was impaired with a high concentration of recombinant CD25 (Fig. 1f, g ), which was consistent with the previous report that soluble CD25 could inhibit the proliferation and function of T cells.^13^ Correlation analysis between sIL-2R concentration and percentage of different immune cell types in COVID-19 patients suggested that sIL-2R might be a negative regulatory factor for T cells, especially CD8+ T cells (Fig. 1h), but not CD4+ T cells, NK cells, and B cells (Supplementary Fig. 1f). The situation of COVID-19 patients is more complicated than we thought. We believe that a high concentration of sIL-2R makes a contribution to lymphopenia in COVID-19, but we do not know whether it is a decisive factor or not. Lymphopenia has been considered to be an indicator of the severity and hospitalization in COVID-19 patients.^2^ The concentration of sIL-2R in the blood may be involved as a co-indicator of the severity in COVID-19 patients with lymphopenia, but more clinical validation with an adequate sample size is required.

In conclusion, we found the concentration of sIL-2R is increasing in COVID-19 patients after illness onset and may be contribute to lymphopenia through IL-2 signaling inhibition. Our results also indicate the potential protective function of IL-2 signaling, which may delay the onset of lymphopenia for COVID-19 patients.

## Supplementary information

Supplemental materials

## References

[1] Cao, X. COVID-19: immunopathology and its implications for therapy. *Nat. Rev. Immunol*. 10.1038/s41577-020-0308-3 (2020). 10.1038/s41577-020-0308-3 PMC7143200 32273594

[2] Tan L (2020). Lymphopenia predicts disease severity of COVID-19: a descriptive and predictive study. Signal Transduct. Target Ther..

[3] Wang, W. et al. High-dimensional immune profiling by mass cytometry revealed immunosuppression and dysfunction of immunity in COVID-19 patients. *Cell. Mol. Immunol.*10.1038/s41423-020-0447-2 (2020). 10.1038/s41423-020-0447-2 PMC7186533 32346099

[4] Hirano, T. & Murakami, M. COVID-19: A new virus, but a familiar receptor and cytokine release syndrome. *Immunity*. 10.1016/j.immuni.2020.04.003 (2020). 10.1016/j.immuni.2020.04.003 PMC7175868 32325025

[5] Zheng, H. Y. et al. Elevated exhaustion levels and reduced functional diversity of T cells in peripheral blood may predict severe progression in COVID-19 patients. *Cell. Mol. Immunol.*10.1038/s41423-020-0401-3 (2020). 10.1038/s41423-020-0401-3 PMC7091621 32203186

[6] Ross SH, Cantrell DA (2018). Signaling and function of interleukin-2 in T lymphocytes. Annu. Rev. Immunol..

[7] Chen, G. et al. Clinical and immunological features of severe and moderate coronavirus disease 2019. *J. Clin. Investig.*10.1172/JCI137244 (2020). 10.1172/JCI137244 PMC7190990 32217835

[8] Gooding R, Riches P, Dadian G, Moore J, Gore M (1995). Increased soluble interleukin-2 receptor concentration in plasma predicts a decreased cellular response to IL-2. Br. J. Cancer.

[9] Lin CY, Lin CC, Hwang B, Chiang BN (1993). Cytokines predict coronary aneurysm formation in Kawasaki disease patients. Eur. J. Pediatr..

[10] Teraura H (2017). The serum concentration of soluble interleukin-2 receptor in patients with Kawasaki disease. Ann. Clin. Biochem..

[11] Sundel RP (2015). Kawasaki disease. Rheum. Dis. Clin. N. Am..

[12] Jones VG (2020). COVID-19 and Kawasaki disease: novel virus and novel case. Hosp. Pediatr..

[13] Schulz O, Sewell HF, Shakib F (1998). Proteolytic cleavage of CD25, the alpha subunit of the human T cell interleukin 2 receptor, by Der p 1, a major mite allergen with cysteine protease activity. J. Exp. Med..

