# Supplementary material for: Potential contribution of increased soluble IL-2R to lymphopenia in COVID-19 patients
Source: Cell Mol Immunol. 2020 Jun 25;17(8):878–80. doi: 10.1038/s41423-020-0484-x (PMC7315399; doi:10.1038/s41423-020-0484-x)

**Potential contribution of increased soluble IL-2R to lymphopenia in COVID-19 patients.**

Yaguang Zhang^*,1^, Xiaojing Wang ^*,2^, Xuezhen Li^*,1^, Dong Xi^*,2^, Ruizhi Mao^3^, Xiaohui Wu^3^, Shipeng Cheng^1^, Xiaoyu Sun^1^,Chunyan Yi^1^, Zhiyang Ling^1^, Liyan Ma^1^, Yiru Fang^#3^ Di Wu ^#2^, Bing Sun ^#,1^, Qin Ning^#,2^

^*^Y.G.Z., X.J.W, X.Z.L. and D.X. contributed equally to this work.

^#^To whom correspondence may be addressed.

^1^State Key Laboratory of Cell Biology, CAS Center for Excellence in Molecular Cell Science, Shanghai Institute of Biochemistry and Cell Biology, Chinese Academy of Sciences, 320 Yueyang Road, Shanghai 200031, China;

^2^Department and Institute of Infectious Disease, Tongji Hospital, Tongji Medical College, Huazhong University of Science and Technology, Wuhan 430030, China.

^3^Clinical Research Center and Division of Mood Disorders, Shanghai Mental Health Center, Shanghai Jiao Tong University School of Medicine, Shanghai, 200030, China.

Corresponding Author

Qin Ning: No. 1095, Jiefang Avenue, Wuhan 430030, China.

Bing Sun: 320 Yueyang Road, Shanghai 200031, China.

Di Wu: No. 1095, Jiefang Avenue, Wuhan 430030, China.

Yiru Fang: 600 Wanping Road, Shanghai 200031, China.

Email addresses of the authors to whom correspondence should be addressed:

[qning@vip.sina.com](mailto:qning@vip.sina.com),

[bsun@sibs.ac.cn](mailto:bsun@sibs.ac.cn),

[woody_1984@163.com](mailto:woody_1984@163.com),

[yirufang@aliyun.com](mailto:yirufang@aliyun.com),

**Contributions**

Q.N, B.S, D.W, and Y.R.F initiated, designed and supervised the study. Y.G.Z and X.Z.L designed and performed in vitro T cell culture. X.J.W, D.X, R.Z.M and X.H.W collected the clinical data. Y.G.Z and B.S analyzed the data and wrote the paper. S.P.C, X.Y.S, C.Y.Y, Z.Y.L, and L.Y.M provided reagents and materials. The authors declare no competing interests.

**Materials and methods**

**Clinical data**

Plasma cytokines including soluble IL-2R (including 3 subunits of IL-2R: CD25, CD122 and CD132), IL-6, IL-10 and TNF-a were measured using the sandwich enzyme-linked immune-sorbent assay (ELISA) method by micro-ELISA autoanalyser (Diasorin Etimax 3000, Germany) for 9 patients according to the manufacturer's instructions. The study protocol for COVID-19 patients was approved and provided by the Institutional Review Board of Tongji Hospital, Tongji Medical College, Huazhong University of Science and Technology (Wuhan, China) (Ethics approval TJ-C20200101). The study protocol for healthy controls was approved and provided by the Shanghai Mental Health Center (Ethics approval 2018-32).

**In vitro cell culture**

Peripheral blood mononuclear cells (PBMC) from healthy donors were activated in a 96-well cell culture plate with human anti-CD3 (5ug/ml, BD) / anti-CD28 (2ug/ml, BD) antibodies and different concentration of recombinant CD25 protein (His Tag, Sino Biological, Cat: 10165-H08H). IL-2 (10U/ml, Protechtech) and IL-2 antibodies (5 ug/ml, eBioscience) were used for the different intensities of IL-2 signaling.

**Flow cytometry analysis of proliferation and function in T cells.**

The cells were stained with antibodies for surface antigens in PBS with 2% FBS. All staining processes were performed according to recommended protocols. Intracellular cytokines staining were performed after 4 hours PMA/Ionomycin re-stimulation. The staining was detected using an Aria II flow cytometer (BD), and the data were analyzed using Flow Jo v10 (BD). The following antibodies were used for staining: Human BD Fc Block(BD, 564220), human CD3 (BD, clone SK7), human CD4 (eBioscience, clone OKT4), human CD8 (BD, clone SK1), human KI67 (Biolegend, clone KI-67), human IFN-gamma (eBioscience, clone GZ-4), human IL-2 (eBioscience, clone MQ1-17H12), Fixable Viability Dyes (BD)

**S-Figure 1. Potential contribution of increased soluble IL-2R to lymphopenia in COVID-19 patients.**

**(A**) Definition of clinical sample collection. (**B**) Percentage of CD4^+^ T cells, CD19^+^ B cells and NK cells in blood from COVID-19 patients within different days after illness onset. The statistical method is the Student's t-test. (**C**) The correlation analysis between TNF-a/IL-6/IL-10 and days after illness onset in COVID-19 patients. The correlation was assessed with Pearson’s test. (**D**) Flow cytometric analysis of KI67 in T cells after anti-CD3 and anti-CD28 antibodies activation for 3 days within low (0.03125ug/ml) or high (8ug/ml) concentration of recombinant CD25 and different intensities of IL-2 signaling. (**E**) Flow cytometric analysis of KI67 in CD3^+^ T cells after anti-CD3 and anti-CD28 antibodies activation for 3 days within 8ug/ml recombinant CD25 and different intensities of IL-2 signaling. **(F**) The correlation analysis between soluble IL-2 receptor (sIL-2R) and cell number/percentage of CD4^+^ T cells, CD19^+^ B cells and NK cells in COVID-19 patients. The correlation was assessed with Pearson’s test.


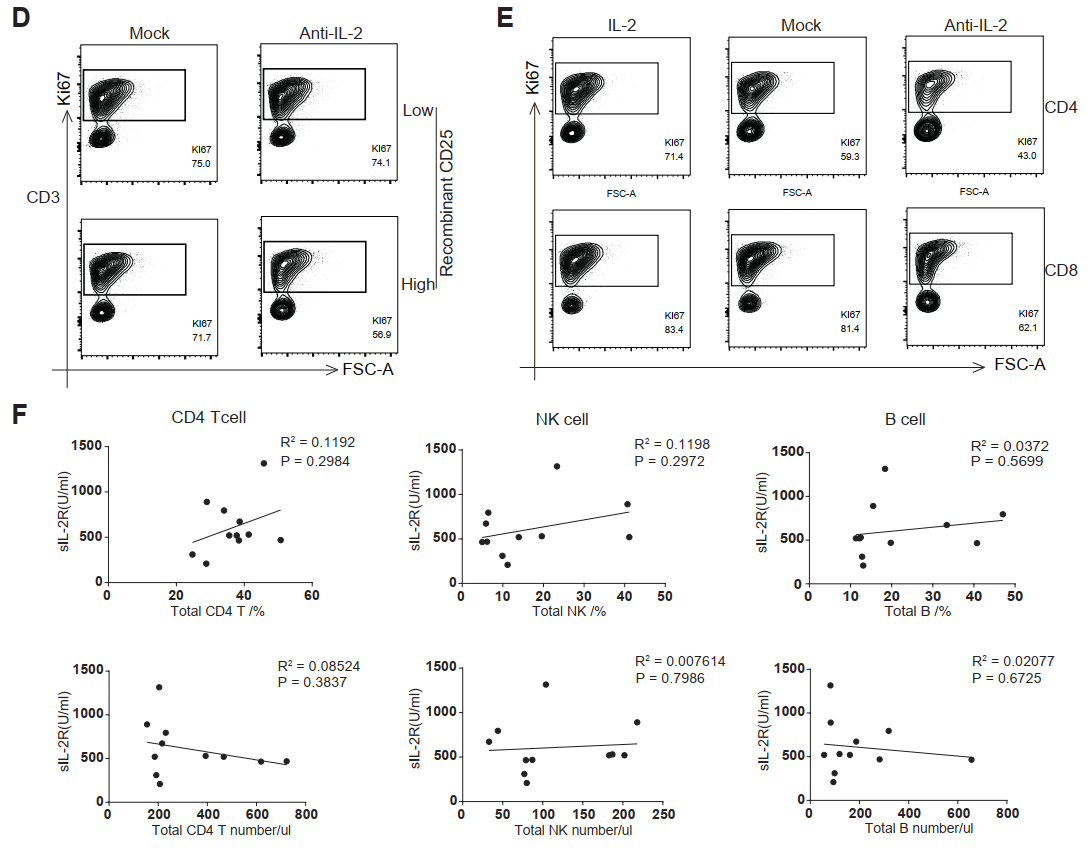

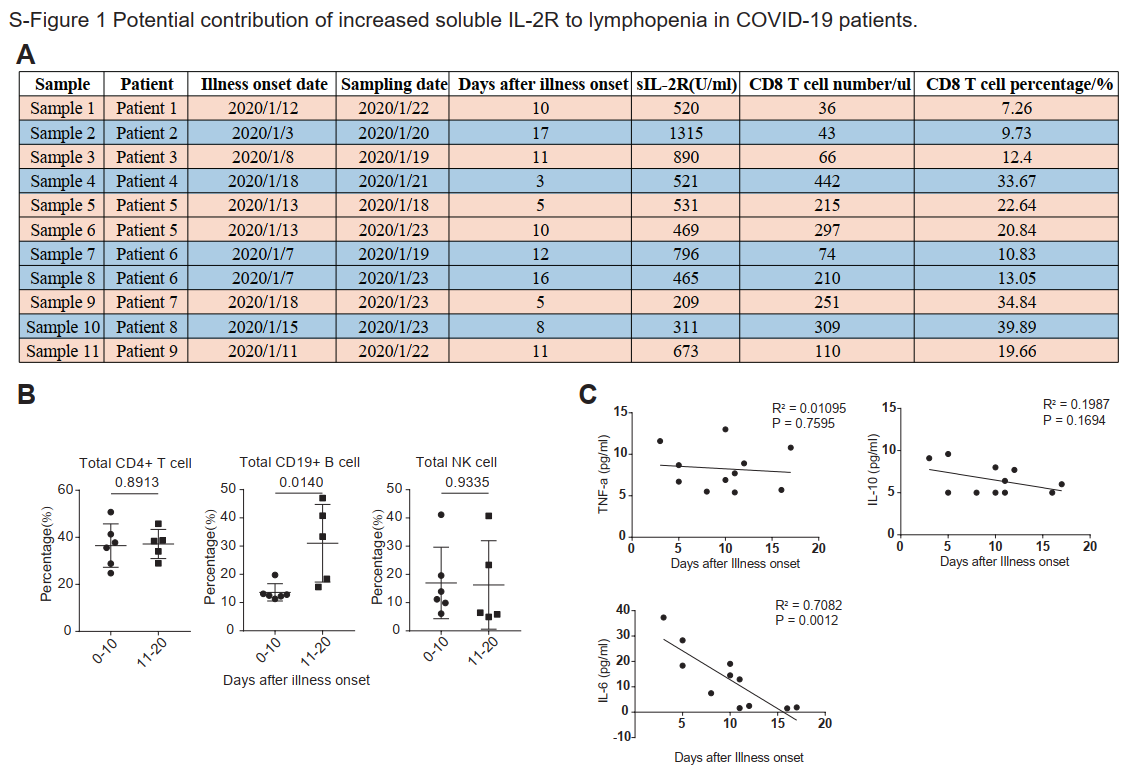

Supplement: Supplementary file 1 — Supplemental materials [file 41423_2020_484_MOESM1_ESM.docx]
